# Cervical Cancer Screening among University Students in South Africa: A Theory Based Study

**DOI:** 10.1371/journal.pone.0111557

**Published:** 2014-11-11

**Authors:** Muhammad Ehsanu Hoque, Shanaz Ghuman, Roger Coopoosmay, Guido Van Hal

**Affiliations:** 1 Graduate School of Business and Leadership, University of KwaZulu-Natal, Durban, South Africa; 2 Department of Environmental Health, Durban University of Technology, Durban, South Africa; 3 Department of Nature Conversation, Mangosuthu University of Technology, Durban, South Africa; 4 Medical Sociology and Health Policy, University of Antwerp, Antwerp, Belgium; Queen Mary Hospital, Hong Kong

## Abstract

**Introduction:**

Cervical cancer is a serious public health problem in South Africa. Even though the screening is free in health facilities in South Africa, the Pap smear uptake is very low. The objective of the study is to investigate the knowledge and beliefs of female university students in South Africa.

**Methods:**

A cross sectional study was conducted among university women in South Africa to elicit information about knowledge and beliefs, and screening history.

**Results:**

A total of 440 students completed the questionnaire. The average age of the participants was 20.39 years (SD  = 1.71 years). Regarding cervical cancer, 55.2% (n = 243) had ever heard about it. Results indicated that only 15% (22/147) of the students who had ever had sex and had heard about cervical cancer had taken a Pap test. Pearson correlation analysis showed that cervical cancer knowledge had a significantly negative relationship with barriers to cervical cancer screening. Susceptibility and seriousness score were significantly moderately correlated with benefit and motivation score as well as barrier score. Self-efficacy score also had a moderate correlation with benefit and motivation score. Students who had had a Pap test showed a significantly lower score in barriers to being screened compared to students who had not had a Pap test.

**Conclusion:**

This study showed that educated women in South Africa lack complete information on cervical cancer. Students who had had a Pap test had significantly lower barriers to cervical cancer screening than those students who had not had a Pap test.

## Introduction

Cervical cancer is a preventable disease through proper screening, treatment and follow-up. But it is a serious public health problem as it accounts for over 275,000 female deaths and approximately 529,000 new diagnoses each year [1]. The World Health Organization (WHO) reported that cervical cancer is the second most common cause of female cancer globally [2]. Over the last three decades, cervical cancer rates have reduced significantly in most of the developed world, because of routine screening programs. In contrast, in most developing countries rates have risen or remained unchanged [3], [4]. While cervical cancer screening has the potential to greatly reduce deaths from cervical cancer, it is a major challenge for developing countries, where lack of resources limits coverage of cervical cancer screening [5]. The HPV prevalence among women in these countries was also relatively higher than in developed regions [6]. Early detection is a proven cost-effective cervical cancer control strategy [7], [8].

Human papillomavirus (HPV) is known to be the main causative agent in cervical cancer. There are over 200 recognized serotypes of the HPV virus. The most common are HPV 16 and HPV 18, which are responsible for approximately 70% of cervical cancer cases. Other factors for increasing young women's vulnerability to cervical dysplasia include oral contraceptive use, smoking, and susceptibility of the adolescent cervix to sexually transmitted infections [9], [10], [11]. Due to the sexually transmitted nature of HPV, early onset of sexual intercourse and multiple sex partners are significant risk factors [12]. It is reported that 80–90% of women will have this sexually transmitted infection at some point in their life, although only 3–4% of them will develop cervical cancer [13], [14].

Young women, especially those of university age, are at higher risk as they tend to be sexually active and have higher numbers of sexual partners [15]. Researchers have reported that young women are poorly informed about cervical cancer and the associated risk factors, are unclear about the purpose of cervical cancer screening, and hold negative or inaccurate beliefs or attitudes about Pap testing [16]–[19].

Current estimates indicate that every year in South Africa, 5743 women are diagnosed with cervical cancer and 3027 die from the disease. It is the second most common cancer among women in South Africa, after breast cancer. It is also the second most frequent cancer among women between 15 and 44 years of age, after breast cancer. At any given time, about 21.0% of women in the general population are estimated to harbor cervical HPV infection in South Africa. It is reported that in South Africa, 62.8% of invasive cervical cancers are attributed to HPVs 16 or 18 [20]. The South African Cancer Association reported that in the year 2006, the age standardised incidence rate for cervical cancer was 24.71 per 100,000 population. The South African Department of Health developed the Cervical Cancer Screening Program which allows three Pap smears per lifetime, at 10 year intervals starting at the age of 30. The target for the policy was the coverage of at least 70% of women nationally [21].

In SA, the cervical cancer screening coverage (proportion of women over the age of 30 years) is low, i.e., 20% nationally [22]. A population-based study conducted among rural South African women reported that only 18% of the women had ever had a Pap smear test [23]. Another study conducted among female university students found that 42.9% of the participants had heard of cervical cancer and only 9.8% of the participants had ever had a Pap smear test [24].

Improving screening services will not of itself be sufficient to result in increased screening uptake, unless we understand and address the multifaceted health beliefs that are likely to influence women's willingness to schedule and obtain screening. Very little is known about South African women's knowledge and beliefs about cervical cancer and screening. No previous study conducted in South Africa investigated women's behavior towards cervical cancer screening. The purpose of this study was to use the Health Belief Model (HBM) to investigate some of the factors influencing women's willingness to schedule and obtain screening. The health impact of college or university students' sexual behavior has been a primary concern, due to their higher levels of sexual experimentation and unsafe sexual practices. The findings of our study will provide insight into the provision of appropriate educational intervention for risk reduction and effective cervical cancer screening uptake among young women in institutions of higher learning.

### HBM and its utilization regarding Pap screening

**Figure 1:**
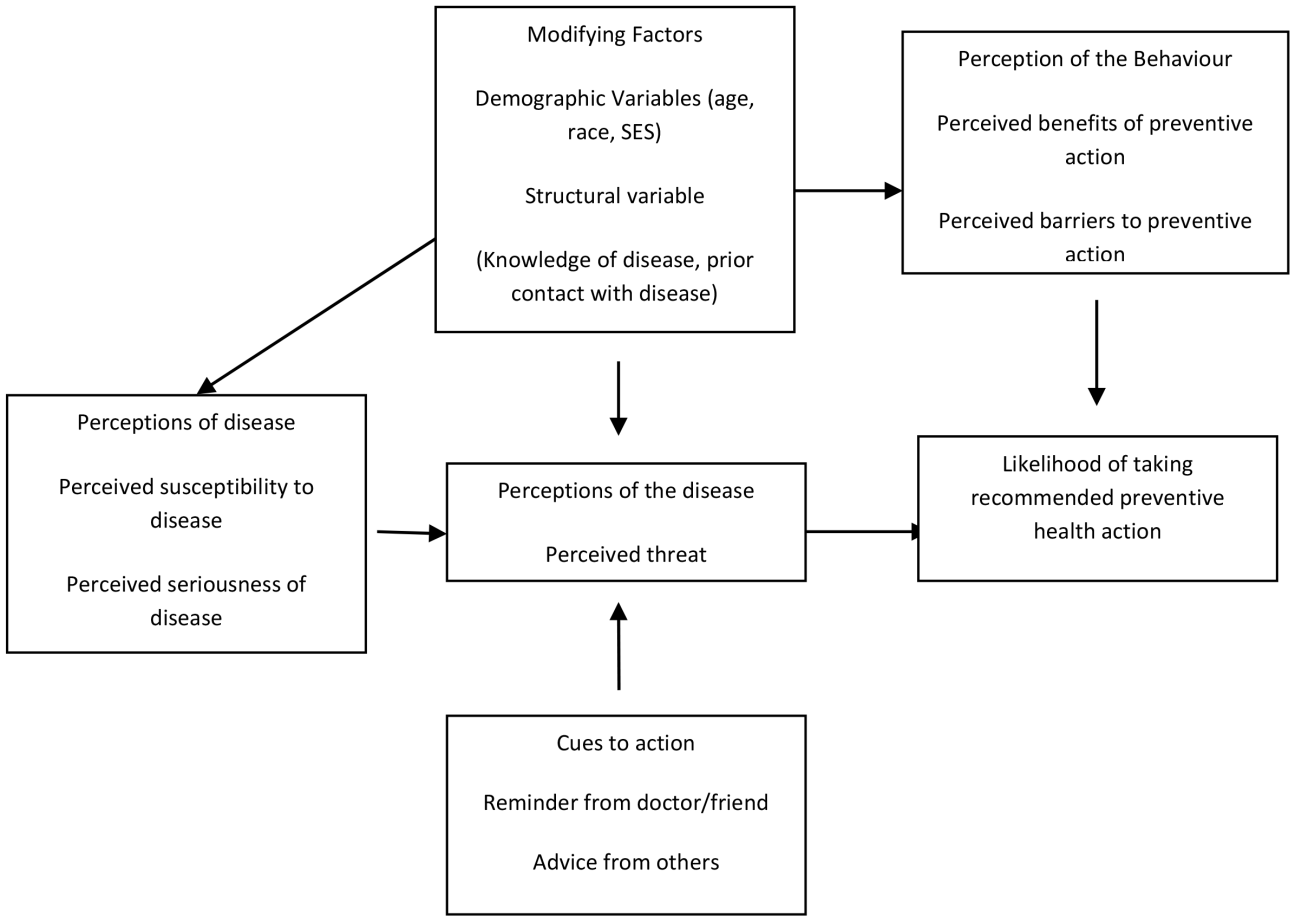
The Health Belief Model (Adapted from Rosenstock, 1974).

According to the HBM (Figure 1), modifying factors, perceptions of the disease, perceptions of behavior, and cues to action simultaneously influence the likelihood of taking a recommended preventive health action [25]. Modifying factors include demographic variables such as age, race, education, income, socio-economic status, psychological variables such as personality type, and lastly, structural variables such as knowledge of the disease and prior contact with the disease. Modifying factors may be understood as either mediating or moderating the relationship between key HBM constructs and the likelihood of taking action.

The key variables in this study are demographic variables, knowledge variables, perceived susceptibility to disease, perceived benefits of cervical cancer screening (CCS), perceived barriers to CCS and the likelihood of CCS uptake. Perceived susceptibility in relation to the HBM indicates that knowledge and awareness about cervical cancer in women may not necessarily result in women attending CCS services. If knowledge of CCS is to be translated into action, (women accepting CCS), each woman must perceive that she is susceptible to developing cervical cancer in her lifetime (perceived susceptibility). Secondly, the women must perceive that cervical cancer is a serious condition (perceived severity of cervical cancer disease) e.g., that cervical cancer is not easily treatable. Thirdly, she must perceive that there are benefits (perceived benefits) to CCS such as early detection and treatment of cervical cancer. Finally, the woman must also perceive that the potential barriers to taking preventive actions, for example costs, are outweighed by potential benefits of taking preventive action, such as early detection and treatment of cervical cancer, which are beneficial for her health and life. The newly amended model would also predict whether a woman is more likely to attend for screening if she is confident that she can do so, and she is motivated to maintain her health.

## Methodology

### Study design and settings

**Figure 2:**
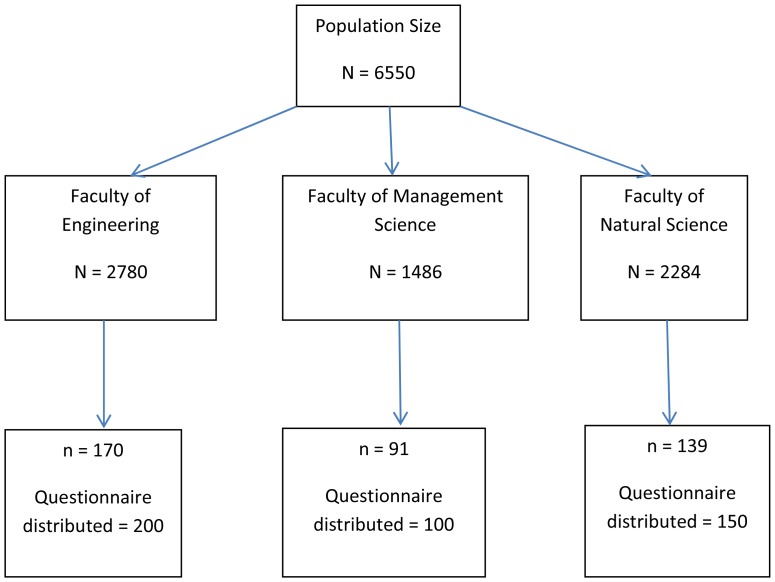
Sample selection.

This was a cross-sectional study conducted amongst female university students who were between the ages of 18 and 26 years. In 2013, a total of 6550 female students were registered. There are three faculties in the university: the faculty of engineering, the faculty of management science and the faculty of natural science. The faculty of engineering had 2780 students, management science had 1486 students and natural science had 2284 students. Using the population size of n = 6550 and a 95% confidence level, the required sample size for the study was n = 364. We added 10% to the sample size for non-response or incompleteness, thus the final sample size for the study became n = 400. The samples were selected using multistage sampling techniques (Figure 2). Firstly, faculties of the university were considered as strata. Then, from each faculty, a number of students were selected based on the proportion of students who were in the faculty according to the year of study. The second and third author went into different classrooms after the lectures were completed and collected the data after prior consultation with the respective lecturers. Those who were present in the classroom were asked to complete the questionnaire. The questionnaires were translated into isiZulu by an isiZulu expert from the university's language center. Informed consent was obtained from participants at the time of completing the questionnaire. The survey was self-administered and anonymous and the completed surveys were anonymously returned to a deposit box on campus. The survey was conducted in March 2013. The research ethics and publication committee of Mangosuthu University of Technology specifically approved this study.

### Instruments

The survey questionnaire has three sections. The first section is related to knowledge, which consists of 13 multiple choice items. Each question has one correct response. HBM construct questions are included in the second section of the questionnaire, and were taken from the ‘Health Belief Model Scale for Cervical Cancer and the Pap Smear Test’ [26]. ‘The Health Belief Model Scale for Cervical cancer and the Pap Smear Test’ has 41 items in six subscales: Susceptibility (1^st^–3^rd^ item), seriousness (4^th^–10^th^ item), benefits/motivation (11^th^–14^th^ and 19^th^–22^th^ item), barriers (23^rd^–38^th^ item), and health motivation (15^th^–17^th^ item). For self-efficacy there are three questions (39^th^–41^st^ item). All the items of the subscale have five-point Likert-type response choices: strongly agree (scores 5 points) to strongly disagree (scores 1 point). Higher scores indicate stronger feeling about that construct. All scales should be positively related to screening behavior except for barriers, which should have a negative association. The last section has 12 questions which focus on sexual behavior and risk factors related to cervical cancer and demographics. These questions are multiple choice. They request information concerning age, marital status, sexual activity, contraceptive behavior, risk factors and past history of Pap smear tests. Three of the questions specifically address preventive behaviors, including number of sexual partners, and Pap smear tests.

### Data analysis

Data were entered into a Microsoft Excel 2003 spreadsheet and imported to SPSS 21.0 version for analysis. The demographics variables are summarized using descriptive summary measures: expressed as mean (standard deviation) for continuous variables, and percent for categorical variables. The Pearson correlation test was carried out between HPV/cervical cancer knowledge, and HBM constructs. The Student t-test was used to compare the mean differences existing in knowledge scores and HBM constructs between women who had had a pap test and those who had not had a Pap test. All statistical tests were performed using two-sided tests at the 0.05 level of significance. P-values were reported to three decimal places with values less than 0.001 being reported as <0.001. P values of less than 0.05 were considered statistically significant.

## Results

A total of 450 questionnaires were distributed, of which 440 questionnaires were completely filled out. The response rate was thus 97.78%. Results indicated that average age of the participants was 20.39 years (SD  = 1.71 years). Only three students were married and the rest were single. Participants' sexual behavior is shown in Table 1. About two-thirds (63%) of the students had had sex before, and among them 77.3% were currently sexually active. The average age of sexual debut among the participants was 18.20 years.

**Table 1:** Sexual orientation of the female university students.

Variables	Frequency	Percent
**Ever had sex before**		
No	163	37.0
Yes	277	63.0
Mean Age at sexual debut	18.20 [Range: 12–22]	
**Presently Sexually Active**		
No	63	22.7
Yes	214	77.3
Average No of sexual partners in lifetime	1.32 [Range: 1–5]	
Use of oral contraceptives (yes)	27	6.1
Smokes cigarettes (n = 440)	37	8.4
**Any family members ever been diagnosed with HPV or CC**	20	4.5

Regarding cervical cancer, 55.2% (n = 243) had ever heard about it and amongst students who had ever had sexual intercourse, 53.2% (147/243) had heard about it. Results indicated that only 15% (22/147) of the students who had ever had sex and had heard about cervical cancer had ever had a Pap test (Data not shown).

Table 2 shows the correlation matrix of cervical cancer knowledge and HBM constructs. Pearson correlation analysis showed that cervical cancer knowledge had a significantly negative relationship with barriers to cervical cancer screening. Results also indicated that susceptible and seriousness score was significantly moderately correlated with benefit and motivation scores as well as barrier score (p<0.01). Self-efficacy score also had a moderate correlation with benefit and motivation score (Table 2).

**Table 2:** Correlation matrix between knowledge and HBM constructs.

	Total Knowledge Score	Total score for susceptibility and seriousness	Benefit and motivation score	Total Barrier score	Self-efficacy score
Total Knowledge Score	1				
Total score for susceptible and seriousness	.013	1			
Benefit and motivation score	−.042	.179**	1		
Total Barrier score	−.224**	.279**	.060	1	
Self-efficacy score	.111	.090	.350**	−.120	1

**. Correlation is significant at the 0.01 level (2-tailed).

The Student t-test was carried out to compare average scores of the HBM constructs between having had a Pap test and not having had a Pap test (Table 3). Results indicated that students who had had a Pap test had a significantly higher average score on knowledge (7.23 vs 5.32), benefit and motivation (33.36 vs 31.02), and self-efficacy (12.45 vs 10.86) compared to students who had not had a Pap test. Regarding barriers to cervical cancer screening, those students who had had a Pap test had significantly lower average scores (36.09 vs 43.71) compared to students who had not had a pap test.

**Table 3:** Comparison of mean knowledge and HBM constructs between students having had a Pap test and not having had a Pap test.

Knowledge and HBM construct	t	p-value	Mean Difference	95% Confidence Interval of the Difference	
				Lower	Upper
Total Knowledge Score	−3.588	.000	−1.95273	−3.02846	−.87699
Total score for susceptibility and seriousness	−1.555	.122	−2.33964	−5.31371	.63444
Benefit and motivation score	−2.152	.033	−3.16218	−6.06705	−.25732
Total Barrier score	4.121	.000	7.62109	3.96629	11.27589
Self-efficacy score	−2.795	.006	−1.59055	−2.71510	−.46600

## Discussion

As far as the authors are aware, this is one of the first studies describing knowledge and beliefs about cervical cancer and screening among a population of university women in South Africa. Overall the study found low awareness levels for the issues related to screening, as there were specific gaps in knowledge about risk factors. This result is not unexpected, given that literate young women in a college environment might have been exposed to public health education messages on sexually transmitted diseases, but mainly HIV/AIDS [27]. A notable finding is that only 21.8% of students knew about HPV. This low level of knowledge has implications for future strategies to prevent cervical cancer with the HPV vaccine. The main cause for concern is that even in these highly educated populations, there is a lack of knowledge about the role of HPV in cervical cancer.

The HBM postulates that people will engage in health seeking behavior if they perceive benefits to themselves accruing from that behavior. The present study found that students who had had a Pap test had significantly higher average scores on benefit and motivation compared to students who had not had a Pap test. This finding is similar to the study conducted among university students in Ghana [27]. The finding is therefore encouraging, and suggests that a program of public education within the context of a national screening program is likely to result in increased screening uptake. Researchers suggest that motivation is the starting point for behavioral performance. They also suggested that behavioral change is most likely when the individual is both motivated to act and has developed strategies and plans which promote behavioral enactment [28].

In the present study, the female students who had heard about cervical cancer and had ever had sexual intercourse had fewer perceived barriers compared to those students who had not had a Pap test. However, subscale scores, including Perceived Seriousness of Cervical Cancer, and Susceptibility to Cervical Cancer, did not differ according to whether or not women had had a Pap test. This finding demonstrates that women do not pay attention to cervical cancer. It also demonstrates that perceived susceptibility to cervical cancer is quite low. A study conducted among women in Turkey also produced similar findings [29]. Researchers reported that South African women do not express all their symptoms when consulting with health care professionals initially [30]. Another South African study concluded that presenting information on cervical cancer in a non-stigmatizing manner, based on the theme of self-protection, promotes cervical screening [31]. A Chinese study reported that women's feelings of uncertainty on receiving an abnormal smear result were mostly related to fear of cancer [32]. Health care workers could provide information to increase knowledge about Susceptibility to Cervical Cancer, Perceived Seriousness of Cervical Cancer and the importance of the Pap Smear Test as a health promotion strategy for all women who have gynecological examination [29].

Women are more likely to engage in health-seeking behavior if they perceive the cost and barriers to such a behavior to be reasonable. This study found a high barrier level among the study participants. For example, fear of a bad result, the test is too painful, partner resisting cervical cancer screening, all suggest that there are cultural and traditional beliefs about societal roles that are influencing these responses. This finding has implications for public health interventions and suggests that broad based public health initiatives will be needed to overcome these barriers. Other important barriers that were mentioned, such as lack of information about cervical cancer, can easily be addressed with simple information provision. Cost barriers were also highlighted but this could be easily resolved as the test is freely available in South Africa. This population also demonstrated some fatalistic beliefs about cancer (*If I am destined to get cancer, I will get it no matter what*). These may be additional cultural barriers to screening rooted in faith that will need to be explored further, and addressed, particularly in older and less educated populations, in whom they may be more prevalent [27].

Our population included young university students, and this has implications for the generalizability of the findings to less educated or older women. The cross sectional nature of the survey means causal inferences cannot be made from the results reported. Furthermore, the survey was self-administered and is therefore open to the usual reporting biases inherent in such surveys. However, we believe that this was minimized because the survey was anonymous. Strengths of the study include the fact that we were able to access a population that has not been widely studied, and that this is one of the first studies describing knowledge and beliefs about cervical cancer in this population and reveals potential targets for interventions to improve cervical cancer screening rates.

## Conclusion

In conclusion, this study showed that a literate population of university women in South Africa lacks complete information on cervical cancer and its risk factors. It was established that cervical cancer knowledge had a significantly negative relationship with barriers to cervical cancer screening. Students who had had a Pap test had significantly higher average scores on knowledge, benefit and motivation, and self-efficacy compared to students who had not had a Pap test. Regarding barriers to cervical cancer screening, those students who had had a Pap test had significantly lower average scores compared to students who had not had a Pap test. In order to influence perceptions, strategies will have to address these barriers by targeting not only the women themselves, but also society at large, and ensure that eligible women receive the right screening cues from both the media and healthcare workers.
